# Sensitization of tamoxifen-resistant breast cancer cells by Z-ligustilide through inhibiting autophagy and accumulating DNA damages

**DOI:** 10.18632/oncotarget.16832

**Published:** 2017-04-04

**Authors:** Hongyi Qi, Zhuyun Jiang, Chengqiang Wang, Yi Yang, Li Li, Hui He, Zanyang Yu

**Affiliations:** ^1^ College of Pharmaceutical Sciences, Southwest University, Beibei District, Chongqing 400716, China

**Keywords:** Z-ligustilide, tamoxifen, autophagic flux, chemoresistance, DNA damage

## Abstract

Autophagy plays a pro-survival role in the tamoxifen-resistant breast cancer cells. Herein we found that autophagy was concomitantly induced in tamoxifen-resistant MCF-7 (MCF-7^TR5^) cells through the dissociation of Bcl-2 from Beclin 1 and subsequent enhancement of interaction among the ATG14-Beclin1-PI3KC3 complex. Moreover, higher level of DNA damage was observed in MCF-7^TR5^ cells with the decreased BRCA1 and RAD51 level and the increased Ku80 level. Interestingly, Nur77 was selectively degraded by autophagy, which causes the release of Ku80 from the Nur77-Ku80 complex, resulting in the increase of the DNA binding of Ku80 and DNA-PKcs. Meanwhile, Z-ligustilide, a phthalide compound from *Radix Angelica sinensis*, was shown to inhibit the autophagic flux by blocking the autophagosome-lysosome fusion. Importantly, Z-ligustilide-mediated autophagy inhibition restored Nur77 expression in MCF-7^TR5^ cells. Furthermore, Z-ligustilide promoted the interaction of Nur77 with Ku80 and thereby abolished the association of DNA-PKcs with DNA ends. Moreover, Z-ligustilide sensitized MCF-7^TR5^ cells in a caspase-independent cell death and enhanced the DNA damage caused by tamoxifen, which was found to be attenuated by shNur77. Together, these findings not only provide important insights into the formation of tamoxifen resistance in breast cancer cells, but also suggest Z-ligustilide may function as a novel autophagy inhibitor to overcome chemoresistance.

## INTRODUCTION

Breast cancer is one of the most frequently diagnosed malignant tumors worldwide among women. In United States, it is estimated that breast cancer makes up 29% of all new cancer diagnoses in women and comprises about 14% of all cancer death among women in 2016 [1]. In China, breast cancer is also the most common malignant tumors in women with approximately 210, 000 new cases diagnosed in women annually. Moreover, it is characterized by an increased incidence and a younger age of onset [2, 3]. Among those patients, approximately 70% of cases belong to estrogen receptor alpha positive (ERα^+^) breast cancer. Thus, the endocrine therapies targeting estrogen and its receptors are an important strategy for breast cancers [4]. Tamoxifen (TAM) is the most widely used endocrine therapy and was first approved by the Food and Drug Administration for the prevention and treatment of ERα^+^ breast cancer [5]. Tamoxifen therapy has been demonstrated to be efficacy in both pre- and post-menopausal women with ERα^+^ breast cancer, leading to almost one-half decrease of the 10-year recurrent risk and approximately one-third decrease of death risk [6]. However, one of the major limitations for tamoxifen is the acquired drug resistance. Although about 70% of ERα^+^ breast cancer patients initially respond to tamoxifen, approximately 50% of them eventually acquire resistance during treatment [7]. Thus, drug resistance remains a major impediment to the successful treatment of breast cancer by tamoxifen.

Autophagy (macroautophagy) is an evolutionarily conserved and highly regulated degradative process activated as an adaptive stress response in unfavorable conditions to maintain homeostasis [8, 9]. Moreover, autophagy is a dynamic process termed “autophagic flux” that comprises five sequential steps: 1) induction or initiation, which involves the formation of a phagophore; 2) nucleation; 3) elongation, which is a critical for the formation of the complete autophagosomes; 4) maturation, which involves the fusion of autophagosomes with lysosomes; and finally 5) degradation, which involves the lysosomal degradation of cytoplasmic proteins, macromolecules, and organelles [8, 10]. Each stage of autophagy is tightly controlled by specific autophagy complexes [11]. Beclin1 plays an important role in both autophagosome formation and autolysosome fusion [12]. The kinase activity of the Beclin1-Vps34 complex is negatively regulated by Bcl-2 family proteins, which bind to Beclin1 and disrupt the interaction between Beclin1 and Vps34, leading to Beclin1 homodimer formation and inhibiting autophagosome formation [13] The role of autophagy in cancer is often reported as confusing and controversial. On the one hand, autophagy has been proposed as a tumor suppressor as defective autophagy is often correlated with malignant transformation and carcinogenesis. One the other hand, autophagy appears to function as a pro-survival and resistance mechanism against cell death caused by cancer therapeutics [14–16]. In the past decade, accumulating evidence demonstrated that autophagy played a pro-survival role in the tamoxifen-resistant breast cancer cells. For instance, the development of tamoxifen-resistant breast cancer cells are accompanied by the induction of autophagy [17]. Meanwhile, autophagy delays apoptotic death in breast cancer cells [18]. Moreover, knockdown of autophagy by pharmacological inhibitors [17, 19] or genetic technique [20, 21] sensitizes breast cancer cells to tamoxifen therapy. Thus, targeting protective autophagy may provide an alternative way to enhance the efficacy of tamoxifen therapy. However, it remains largely unknown the exact mechanisms responsible for the formation of protective autophagy and how autophagic flux renders the resistance of breast cancer cells to TAM.

*Radix Angelica sinensis* is the dried root of Angelica sinensis (Oliv.) Diels (Apiaceae) and used as a medicinal herb in China for gynecological disorders with a history of over two thousand years. Recent investigation demonstrated that *Radix Angelica sinensis* is most frequently prescribed in herbal formula for breast cancer in Taiwan [22]. Furthermore, *Radix Angelica sinensis* is regularly taken by almost half of tamoxifen-treated breast cancer survivors [23]. Z-ligustilide (Z-LIG) is a representative phthalide compound and accounts for more than 50% in the volatile oil of *Radix Angelica sinensis* [24]. Recently, Z-LIG was shown to exert inhibitory effect on a variety of human tumors, including colorectal cancer [25] and prostate cancer [26], leukemia [27] and brain tumor [28]. However, the effect of Z-LIG on breast cancer remains unknown. Notably, Z-LIG has been observed to inhibit tumor necrosis factor-alpha-induced autophagy during C2C12 cells differentiation [29]. However, the exact role of Z-LIG on the autophagic flux is still largely unclear. Moreover, it's very interesting to us that whether Z-LIG could inhibit the protective autophagy in tamoxifen-resistant breast cancer cells and thereby enhance the efficacy of tamoxifen therapy.

In this study, we first determined whether the change of interaction between Bcl-2 and Beclin 1 was responsible for the formation of protective autophagy in the established TAM-resistant breast cancer cells. Then, we characterized the Z-LIG-mediated autophagy inhibition and the underlying mechanisms. Moreover, the level of DNA damage and the DNA repair mechanisms in TAM-resistant breast cancer cells were examined. Furthermore, the correlation of protective autophagy and the change of DNA repair mechanisms was also determined. Finally, the effect of Z-LIG-mediated autophagy inhibition on the DNA damage and the DNA repair mechanism in TAM-resistant breast cancer cells was specially examined.

## RESULTS

### Dissociation of Bcl-2 from Beclin 1 concomitantly confers protective autophagy in MCF-7^TR5^ cells

**Figure 1:**
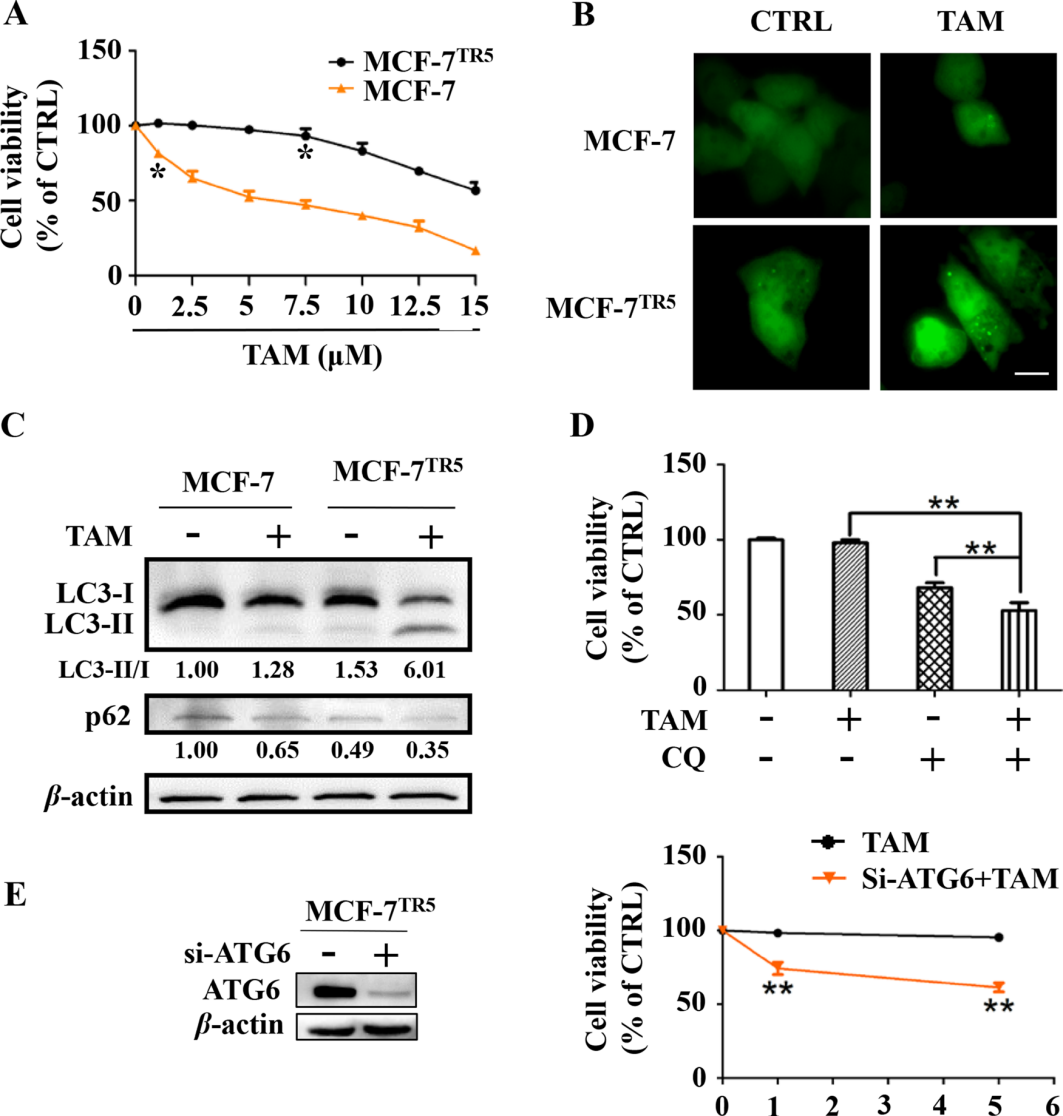
Protective autophagy is concomitantly activated in MCF-7TR5 cells (**A**) Effect of TAM on the cell viability of MCF-7 and MCF-7^TR5^ cells. Cells were treated by TAM as indicated for 72 h and cell viability were determined by SRB assay. (**B**) GFP-LC3 punctation in MCF-7 and MCF-7^TR5^ cells in the presence and absence of TAM. Cells were transfected with GFP-LC3 for 6 h and then treated with TAM (5 μM) for 12 h. A punctate distribution of LC3 in both cells was observed by confocal microscopy (40×). Scale bars: 10 μm. (**C**) Conversion of LC3II and p62 expression in MCF-7 and MCF-7^TR5^ cells. Cells were treated with or without TAM (5 μM) for 12 h, respectively. Then, LC3II/I and p62 expression was determined by Western blotting. (**D**) MCF-7^TR5^ cells were pretreated with or without autophagy inhibitor CQ (10 μM) for 12 h and then treated with TAM (5 μM) for 72 h. The cell viability was determined by SRB assay. (**E**) MCF-7^TR5^ cells were transfected with siATG6 or siCTRL for 6 h, and then treated with TAM (0, 1, 5 μM) for 48 h. The cell viability was determined by SRB assay. The blots were a representative of three independent experiments. Values represent mean ± SD. *p < 0.05, **p < 0.01 vs. control or as indicated.

In the current study, we first established the stable TAM-resistant cell models for ERα^+^ breast cancer cells. A stepwise drug selection was used to generate TAM-resistant breast cancer cells, named MCF-7^TR5^ or T47D^TR5^ (TAM resistant to 5 μM). To verify the efficacy of these established models, we compared the cytotoxicity of TAM to both sensitive and resistant ERα^+^ breast cancer cells. As a result, TAM caused dose-dependent cell death in both MCF-7 and T47D cells and only 1 μM of TAM already caused significant cell death (*p* < 0.05). However, TAM exhibited only weak inhibitory effect on both MCF-7^TR5^ and T47D^TR5^ and significant cell death induced by TAM was not observed until 7.5 μM (*p* < 0.05) (Figure 1A and Supplementary Figure 1). Previous study demonstrated that TAM-resist ERα^+^ breast cancer cells was accompanied by autophagy [17]. We thereby compared the autophagy induced by TAM between drug-resistant cell lines and wide-type cell lines. First, we examined the changes of the GFP-LC3 distribution pattern in MCF-7 and MCF-7^TR5^ cells with transient expression of the GFP-LC3, which is a well-known fluorescent marker of autophagosome. As shown in Figure 1B, the GFP-LC3 puncta in MCF-7^TR5^ cells was more than that in MCF-7 cells, and TAM further enhanced the GFP-LC3 punctation in MCF-7^TR5^. Then, we also checked the changes of LC3 conversion and the level of p62 in both MCF-7 and MCF-7^TR5^ cells by Western blotting. The conversion of LC3-I to LC3-II was obviously enhanced and the expression of p62 was significantly decreased in MCF-7^TR5^ cells compared with those in MCF-7 cells. Moreover, TAM dramatically promoted these changes (Figure 1C). In addition, we attempted to determine whether the autophagy induced by TAM serves as a pro-survival or pro-death mechanism. We used chloroquine (CQ), a well-characterized autophagy inhibitor, to inhibit autophagy and checked its effect on MCF-7^TR5^ cells. As shown in Figure. 1D, TAM (5 μM) alone showed no cytotoxicity to MCF-7^TR5^ cells and CQ caused moderate cytotoxicity (*p* < 0.01), while TAM combined with CQ markedly decreased the cell viability of MCF-7^TR5^ cells compared with each alone (*p* < 0.01). Then, we further verified the role of autophagy in cell death by manipulating the autophagy level via siATG6. We found that suppression of autophagy by siATG6 remarkably sensitized MCF-7^TR5^ cells to both 1 μM and 5.0 μM of TAM (*p* < 0.01) (Figure 1E). Thus, autophagy serves as a pro-survival mechanism in MCF-7^TR5^ cells.

**Figure 2:**
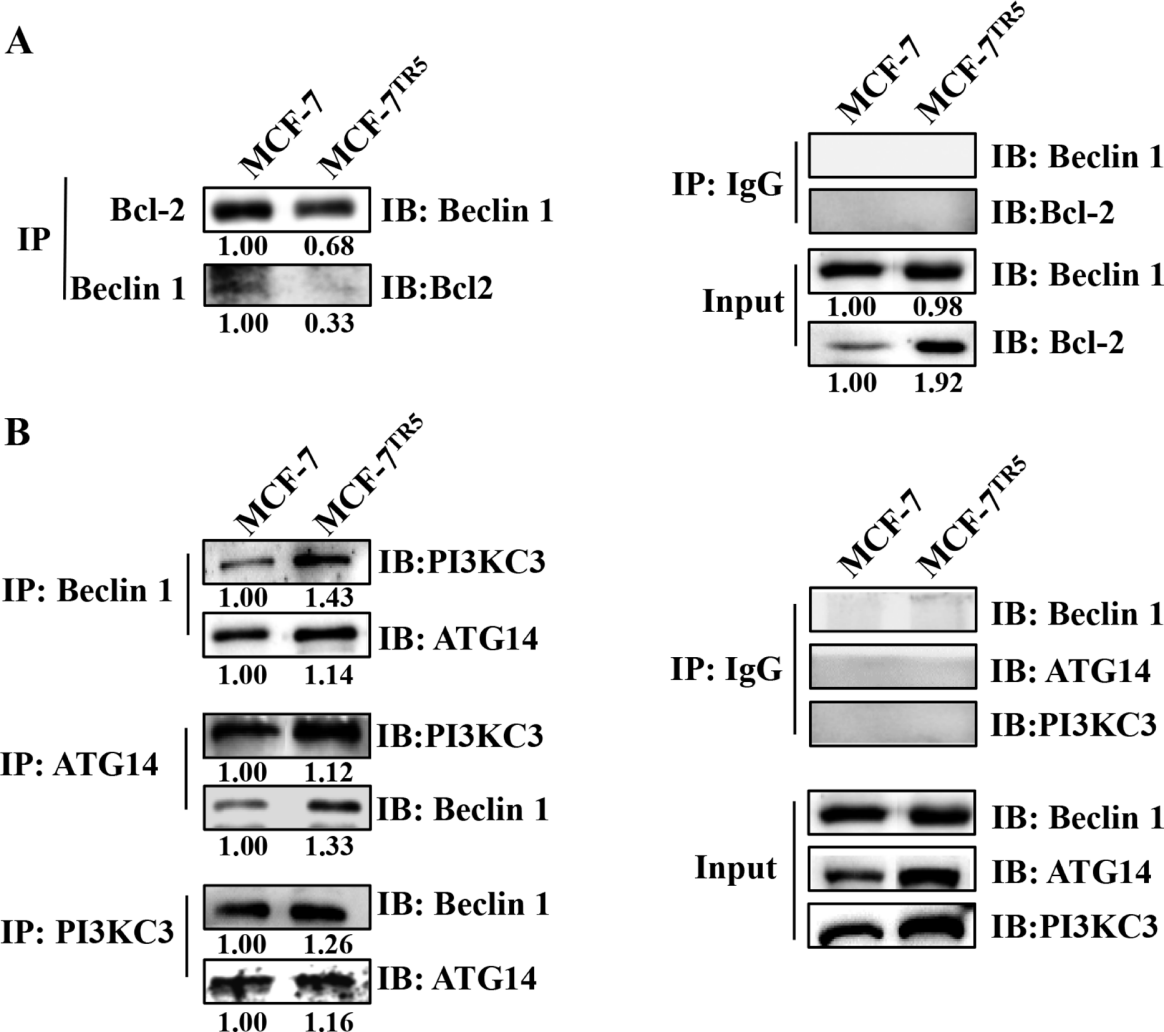
Dissociation of Bcl-2 from Beclin 1 confers protective autophagy in MCF-7TR5 cells Immunoprecipitation assay of Bcl-2-Beclin1 interaction (**A**) and ATG14-Beclin1-PI3KC3 interaction (**B**) in MCF-7 and MCF-7^TR5^ cells. Cell lysate was prepared and subjected to immunoprecipitation using anti-Bcl-2, anti- Beclin1 or anti-Beclin1, anti-ATG14, anti-PI3KC3, and then the expression of the associated Bcl-2, Beclin1, ATG-14 and PI3KC3 was determined using immunoblotting, respectively.

To clarify why the autophagy level was higher in MCF-7^TR5^ cells, we compared the possible difference of the interaction between Beclin1 and Bcl-2, Class III PI3K (PI3KC3) or ATG14, which is known to mediate the formation of autophagosome, using co-immunoprecipitation assays in both MCF-7 and MCF-7^TR5^ cells. Our findings showed that the binding of Bcl-2 to Beclin1 was significantly decreased in MCF-7^TR5^ cells (Figure 2A), whereas the interactions among Beclin1, ATG14 and PI3KC3 was markedly enhanced in MCF-7^TR5^ cells compared with those in MCF-7 cells (Figure 2B).

### Z-LIG functions as autophagy inhibitor in MCF-7^TR5^ cells

**Figure 3:**
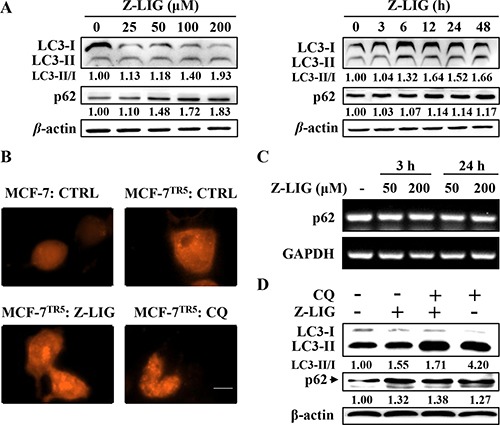
Z-LIG functions as autophagy inhibitor in MCF-7TR5 cells (**A**) Effect of Z-LIG on conversion of LC3II and p62 expression. MCF-7^TR5^ cells were treated with Z-LIG with indicated concentrations for 24 h (left panel) or treated with Z-LIG (50 μM) for indicated time points (right panel). Then, the expression of LC3II/I and p62 was analyzed by Western blotting. (**B**) Effect of Z-LIG on RFP-LC3 punctation. Cells were transfected with RFP-LC3 for 6 h, and then MCF-7^TR5^ cells were treated with Z-LIG (50 μM) and CQ (20 μM) for 24 h. A punctate distribution of LC3 was observed by confocal microscopy (40×). Scale bars: 10 μm. (**C**) Effect of Z-LIG on p62 mRNA expression. MCF-7 cells were treated with Z-LIG as indicated for 3 or 24 h, then expression of p62 mRNA was determined by RT-PCR. (**D**) Effect of combinatorial Z-LIG and CQ on conversion of LC3II and p62 expression. Cells were treated with CQ (20 μM) or Z-LIG (50 μM) or their combination for 24 h. And then the expression of LC3II/I and p62 was analyzed by Western blotting. The blots were a representative of three independent experiments.

To identify the effect of Z-LIG on autophagy, we first examined the influence of Z-LIG on conversion of LC3-I to LC3-II in MCF-7^TR5^ cells. Western blotting analysis showed that Z-LIG treatment resulted in concentration- and time-dependent increase of LC3-II/LC3-I ratio in MCF-7^TR5^ cells (Figure 3A). Next, we further determined the changes of the RFP-LC3 distribution pattern in both MCF-7 and MCF-7^TR5^ cells. Consistent with the result in Figure 1B, MCF-7^TR5^ cells had a more accumulation of RFP-LC3 puncta compared with MCF-7 cells. Importantly, the addition of Z-LIG further increased the RFP-LC3 punctation, which is similar with the incorporation of CQ (Figure 3B). Since the accumulation of LC3-II level and RFP-LC3 puncta may represent either the increased generation of autophagosomes or a blockage in autophagosomal maturation and degradation. Therefore, we investigated the change of autophagic flux by Z-LIG. As shown in Figure 3A, we simultaneously tracked the p62 protein level, which binds to LC3 and degraded by autophagy. Z-LIG could significantly enhance the p62 protein level in both concentration- and time-dependent manners in MCF-7^TR5^ cells. Moreover, the increased p62 protein level was also observed in T47D^TR5^ cells (Supplementary Figure 2). Similarly, we also found that Z-LIG increased the LC3-II/LC3-I ratio and p62 protein level in U251 cells and MDA-MB-231 cells (Supplementary Figure 3). Then, we determined whether Z-LIG-induced p62 protein accumulation is due to transcriptional activation using RT-PCR assay. The result showed that Z-LIG treatment did not alter p62 mRNA levels (Figure 3C), suggesting that Z-LIG is capable of decreasing autophagic flux in MCF-7^TR5^ cells. Furthermore, the effect of Z-LIG on autophagy flux was identified by treating cells with CQ. As shown in Figure 3D, both Z-LIG and CQ alone caused the increase of LC3-II/LC3-I ratio and p62 level. Notably, no further increase of LC3-II/LC3-I ratio and p62 level was observed after combining Z-LIG with CQ, which indicates that Z-LIG is also a potent autophagy flux inhibitor as CQ.

### Z-LIG blocks autophagosome-lysosome fusion and impairs lysosomal activity

**Figure 4:**
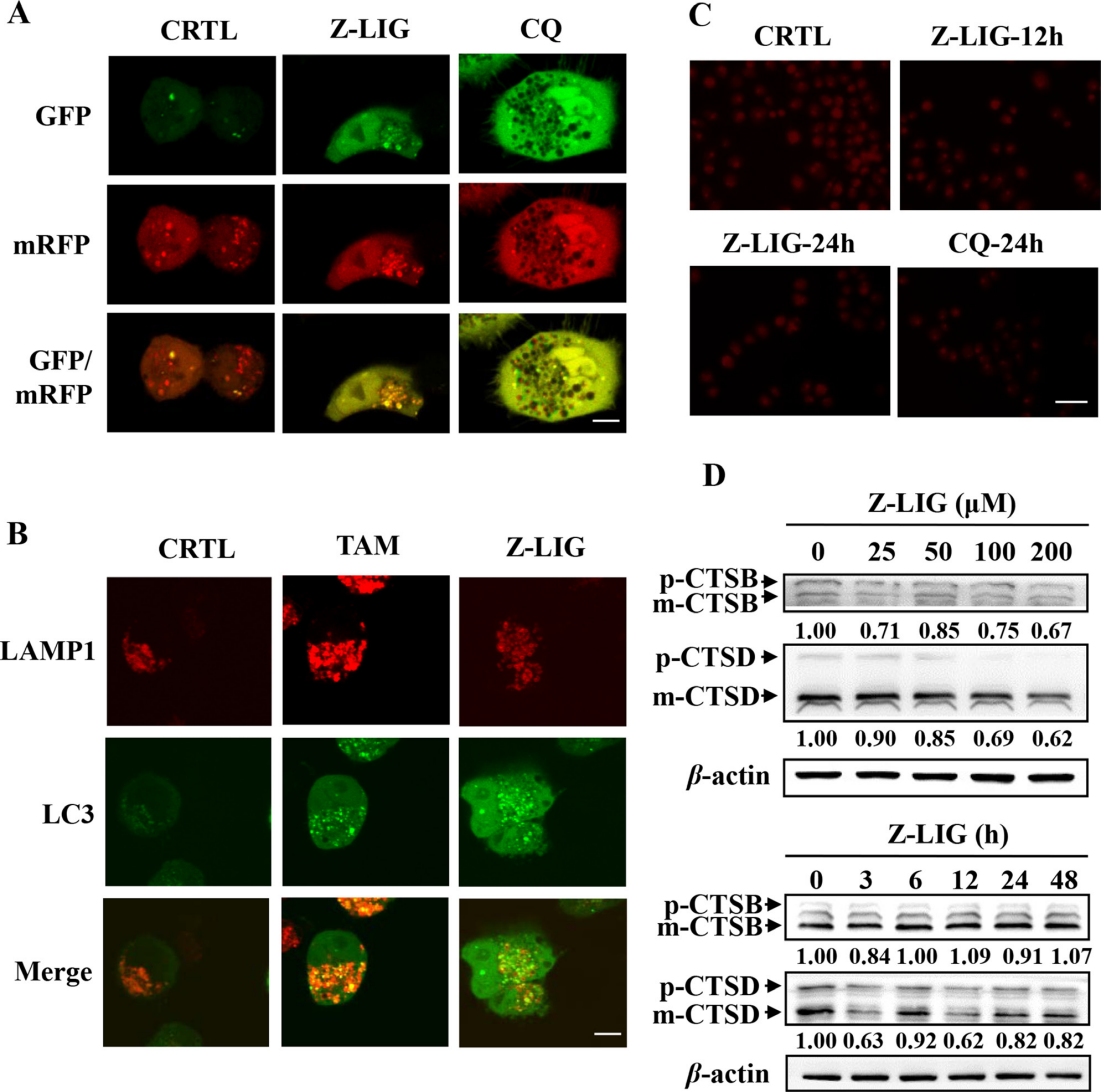
Z-LIG blocks autophagosome-lysosome fusion and impairs lysosomal activity (**A**) MCF-7^TR5^ cells were transfected with a tandem reporter construct mRFP-GFP-LC3(tfLC3) for 6 h, and then exposed to Z-LIG (50 μM) and CQ (20 μM) for 24 h. The colocalization of GFP and mRFP-LC3 puncta was examined by confocal microscopy. Scale bars: 10 μm. (**B**) MCF-7^TR5^ cells were co-transfected with LAMP1-mRFP and GFP-LC3 for 6 h, and then exposed to Z-LIG (50 μM) and TAM (5 μM) for 24 h. The colocalization of LC3 and LAMP1 was examined by confocal microscopy. Scale bars: 10 μm. (**C**) Z-LIG affects AO staining of acidic compartments. MCF-7^TR5^ cells were treated with Z-LIG (50 μM) for 12 or 24 h and CQ (20 μM) for 24 h, respectively. Then, cells were stained by AO and observed under fluorescence microscope (200×). Scale bars: 100 μm. (**D**) MCF-7^TR5^ cells were treated with Z-LIG as indicated for 24 h (above panel) or treated with Z-LIG (50 μM) for indicated time points (below panel). And the expression of CTSB and CTSD was determined by Western blotting. The blots were a representative of three independent experiments.

To address whether Z-LIG affect autophagosome-lysosome fusion, we transfected an mRFP-GFP-LC3 construct (a tandem fluorescent-tagged LC3 reporter containing monomeric red fluorescent protein (mRFP) and GFP; tfLC3) into MCF-7^TR5^ cells to observe the autophagosome maturation and autolysosome formation. Similar with CQ, treatment with Z-LIG induced both GFP and mRFP puncta formation and colocalization, whereas red LC3 puncta was abundant in MCF-7^TR5^ cells treated by only vehicle (Figure 4A). Then, we further checked the autophagosome-lysosome fusion process by tracking the late lysosome marker LAMP-1 to monitor its colocalization with the autophagosomal marker GFP-LC3 in MCF-7^TR5^ cells. As shown in Figure 4B, the GFP-LC3 punctation in MCF-7^TR5^ cells treated by Z-LIG was not colocalized with LAMP-1. In contrast, there was extensive colocalization of GFP-LC3 and LAMP-1 in MCF-7^TR5^ cells treated by TAM. These findings raised the possibility that the Z-LIG may inhibit autophagy flux by interfering fusion of autophagosome with lysosome. Next, we attempted to examine the effect of Z-LIG on lysosomal function. As the enzymatic activity of cathepsins in lysosome functions optimally over a narrow range of acidic pH values, we firstly used the acridine orange (AO), a nucleic acid dye that accumulates in acidic spaces, to evaluate the lysosomal pH. As shown in Figure 4C, unlike the CTRL, the red fluorescent signal was greatly reduced in both Z-LIG- and CQ-treated MCF-7^TR5^ cells. Cathepsins as the major lysosomal proteases play important role in maintaining cellular homeostasis and differentiation by recycling cellular contents [30]. We thereby evaluated whether Z-LIG affected the maturation of lysosomal cathepsins, CTSB and CTSD. As a result, Z-LIG caused a concentration- and time-dependent downregulation of CTSD protein level of both precursors and mature forms in MCF-7^TR5^ cells (Figure 4D), whereas no obvious decline was observed in the CTSB protein level. CTSD protein level also decreased in T47D^TR5^ cells (Supplementary Figure 4). In addition, Z-LIG also resulted in a significant reduction of CTSD mRNA expression as revealed by RT-PCR assay (Supplementary Figure 5). Collectively, these results demonstrate that Z-LIG has an adverse effect on lysosomal function by altering lysosomal pH and downregulating lysosomal cathepsins.

### Z-LIG sensitizes TAM-resistant breast cancer cells to apoptosis in a caspase- independent manner

**Figure 5:**
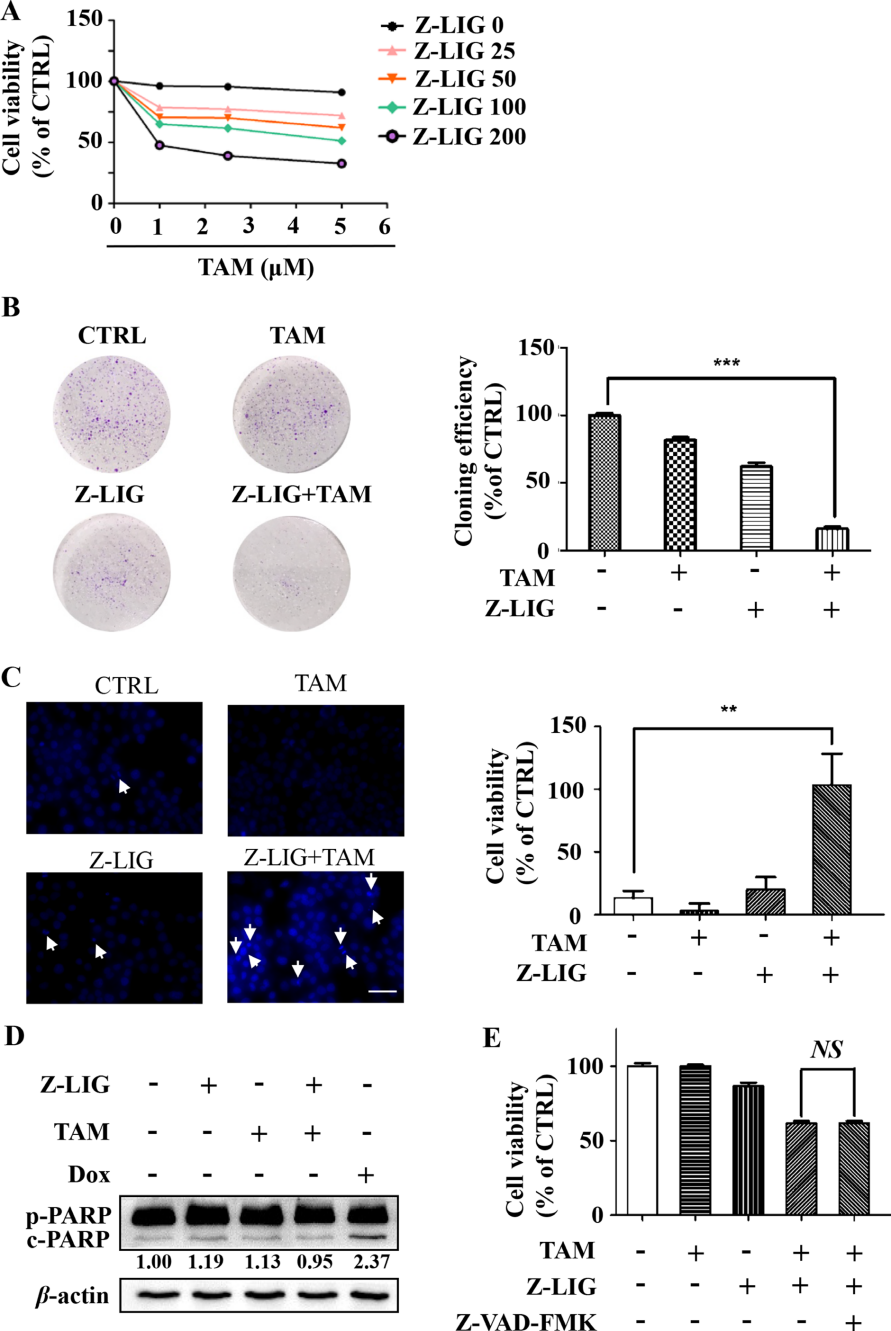
Z-LIG sensitizes TAM-resistant breast cancer cells in a caspase- independent manner (**A**) Effect of combinatorial Z-LIG and TAM on cell viability. MCF-7^TR5^ cells were pretreated with Z-LIG as indicated for 12 h and then treated with TAM as indicated for 72 h. The cell viability was determined by SRB assay. (**B**) Effect of combinatorial Z-LIG and TAM on colony formation. MCF-7^TR5^ cells were pretreated with or without Z-LIG (50 μM) for 12 h, then treated with TAM (2.5 μM) and allowed to grow for 2 weeks before stained with 0.005% crystal violet. Adjacent picture depicts the crystal violet-stained colonies and bar graph indicated the cloning efficiency compared with untreated control. (**C**) MCF-7^TR5^ cells were pretreated with or without Z-LIG (50 μM) for 12 h and then exposed to TAM (5 μM) for 48 h. Morphologic change of apoptotic cells was evaluated by Hoechst 33342 staining and observed by fluorescence microscope (400×). Scale bars: 100 μm. (**D**) MCF-7^TR5^ cells were pretreated with or without Z-LIG (50 μM) for 12 h, and then were exposed to TAM (5 μM) or DOX (0.5 μM) as positive control. The expression of pro-PARP (p-PARP) and cleaved-PARP (c-PARP) were determined by Western blotting. The blots were a representative of three independent experiments. (**E**) MCF-7^TR5^ cells were pretreated with or without pan-caspase inhibitor Z-VAD-FMK (25 μM) for 2 h and with or without Z-LIG (50, 200 μM) for 12 h, and then treated with TAM (5 μM). The cell viability was determined by SRB assay. Values represent mean ± SD. NS, non-significant. Values represent mean ± SD. **p < 0.01. ***p < 0.001.

Our data suggest that autophagy serves as a pro-survival function in MCF-7^TR5^ cells (Figure 1), whereas Z-LIG can mediate autophagy inhibition and lysosomal dysfunction (Figures 3 and 4). It is interesting to us that whether the suppression of autophagic flux by Z-LIG sensitized MCF-7^TR5^ cells to TAM. Thus, we used Z-LIG to inhibit autophagy and tested its effect on TAM-induced cell death. Firstly, SRB assay was performed to determine the cell viability. Treatment with TAM alone (1~5 μM) showed non-cytotoxicity to MCF-7^TR5^ cells, whereas nearly 40% decrease of the cell viability was detected from TAM (5 μM) combined treatment with low concentration (50 μM) of Z-LIG, and more than 70% of the cells were killed by TAM (5 μM) in presence of a high concentration of Z-LIG (200 μM) (Figure 5A). Similar results were also observed in T47D^TR5^ cells (Supplementary Figure 6). Furthermore, the colony formation assay was performed to determine the effect of long-term treatment on the cell growth. Although Z-LIG or TAM alone could reduce the number of colony formation to a certain extent, combined treatment with Z-LIG (50 μM) and TAM (2.5 μM) almost completely suppressed colony formation of MCF-7^TR5^ cells (*p* < 0.001) (Figure 5B). These results indicate that MCF-7^TR5^ cells with compromised autophagic flux after Z-LIG treatment show higher sensitivity to TAM. Then, we determined the apoptosis by Hoechst 33342 staining in MCF-7^TR5^ cells. As shown in Figure 5C, the combinatorial Z-LIG (50 μM) and TAM (5 μM) exhibited much more cells with condensed and fragmented nuclei than control (*p*<0.01). To further characterize the apoptosis-inducing effect, we determined the apoptosis-related protein in MCF-7^TR5^ cells by Western blotting. Figure 5D showed that PARP cleavage was not promoted by the combination of Z-LIG and TAM in MCF-7^TR5^ cells, whereas doxorubicin (Dox) as positive control clearly led to PARP cleavage. Moreover, when MCF-7^TR5^ cells were co-treated with a general caspase inhibitor (Z-VAD-FMK), no influence was observed on the cell death induced by the combination of Z-LIG and TAM (Figure 5E). Therefore, these results suggest that Z-LIG promotes caspase-independent cell death in TAM-resistant breast cancer cells.

### MCF-7^TR5^ cells exhibited higher DNA damage level and impaired DSB repair mechanism

**Figure 6:**
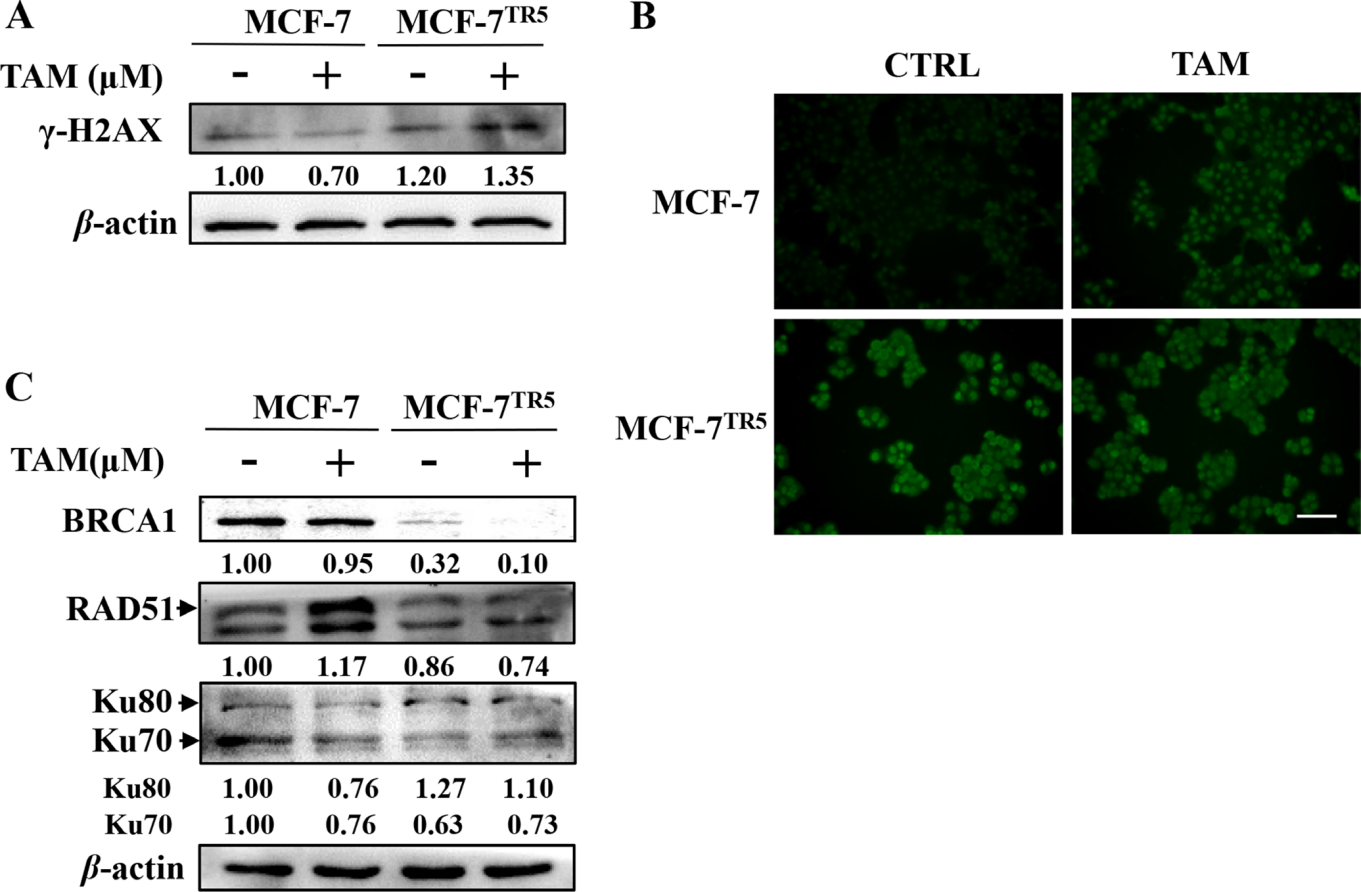
Higher DNA damage level and impaired DSB repair mechanisms were observed in MCF-7^TR5^ cells (**A**) Expression of γ-H2AX in MCF-7 and MCF-7^TR5^ cells. Cells were treated with or without TAM (5 μM) for 12 h, respectively. Then, expression of γ-H2AX was determined by Western blotting. The blots were a representative of three independent experiments. (**B**) Immunofluorescence staining of γ-H2AX in MCF-7 and MCF-7^TR5^ cells. Cells were treated with TAM (5 μM) for 12 h, respectively. Immunofluorescence staining of γ-H2AX was observed by fluorescence microscope (400×). Scale bars: 100 μm. (**C**) Expression of BRCA1, RAD51 and Ku80/Ku70 in MCF-7 and MCF-7^TR5^ cells. Cells were treated with TAM (5 μM) for 12 h, respectively. Then, expression of BRCA1, RAD51 and Ku80/Ku70 were determined by Western blotting. The blots were a representative of three independent experiments.

Change of DNA damage were then determined in MCF-7 and MCF-7^TR5^ cells. As a result, higher level of γ-H2AX, a DNA double-strand break (DSB) marker, was observed in MCF-7^TR5^ cells whether TAM (5 μM) treatment or not compared with that in MCF-7 cells (Figure 6A). And we further examined the accumulation of γ-H2AX by immunofluorescence. Consistently, more green fluorescence of γ-H2AX was observed in MCF-7^TR5^ cells (Figure 6B). These results indicate DNA damage was more serious in MCF-7^TR5^ cells. Then, we also examined the level of proteins associated with DNA damage repair pathway. As shown in Figure 6C, the level of BRCA1, a protein playing a central role in homologous recombination (HR) repair mechanism [31], dramatically decreased in MCF-7^TR5^ cells. Moreover, the level of RAD51, which is recruited by BRCA1 to induce DNA damage repair [31], also reduced in MCF-7^TR5^ cells. However, the level of Ku80 (but not Ku70), a major player in the non-homologous end-joining (NHEJ) repair mechanism [31], obviously increased in MCF-7^TR5^ cells.

### Restoration of autophagy-degraded Nur77 by Z-LIG is essential for DNA damage and cell death caused by combinatorial Z-LIG and TAM

**Figure 7:**
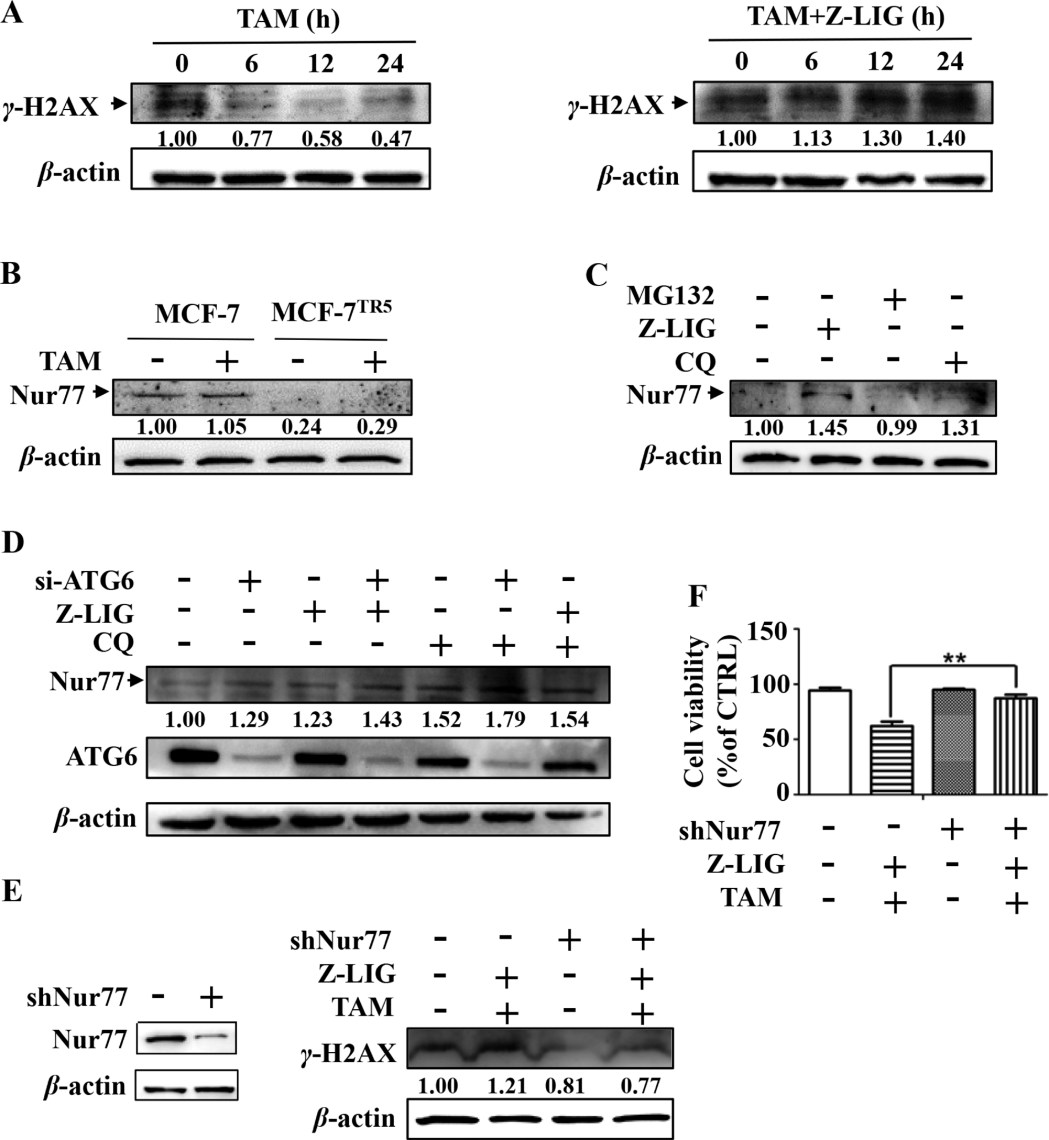
Restoration of autophagy-degraded Nur77 by Z-LIG is essential for DNA damage and cell death caused by combinatorial Z-LIG and TAM (**A**) MCF-7^TR5^ cells were pretreated with or without Z-LIG (50 μM) for 12 h and then exposed to TAM (5 μM) for 0, 6, 12 and 24 h. The expression of γ-H2AX was determined by Western blotting. The blots were a representative of three independent experiments. (**B**) Expression of Nur77 in MCF-7 and MCF-7^TR5^ cells. Cells were treated with or without TAM (5 μM) for 12 h, respectively. Then, expression of Nur77 was determined by Western blotting. The blots were a representative of three independent experiments. (**C**) MCF-7^TR5^ cells were exposed to MG132 (5 μM), Z-LIG (50 μM) and CQ (20 μM) for 6 h, respectively. Then, the expression of Nur77 was determined by Western blotting. The blots were a representative of three independent experiments. (**D**) MCF-7^TR5^ cells were transfected with siATG6 or siCTRL for 6 h, and then treated with or without Z-LIG (50 μM) or CQ (20 μM) for 24 h. Then, the expression of Nur77 and ATG6 were determined by Western blotting. The blots were a representative of three independent experiments. (**E**) MCF-7^TR5^ cells were transfected with shNur77 or sh-NC for 6 h and pretreated with or without Z-LIG (50 μM) for 12 h, followed by with or without TAM (5 μM) for 24 h. The expression of γ-H2AX was determined by Western blotting. The blots were a representative of three independent experiments. (**F**) MCF-7^TR5^ cells were treated as (E). And then the cell viability was determined by SRB assay. Values represent mean ± SD. ^*^p < 0.01.

To evaluate the influence of Z-LIG on TAM-induced DNA damage, we treated MCF-7^TR5^ and T47D^TR5^ cells with TAM alone or combinatorial TAM and Z-LIG for different time-points. As a result, γ-H2AX levels gradually decreased in TAM alone-treated MCF-7^TR5^ (Figure 7A) and T-47D^TR5^ cells (Supplementary Figure 7) as a result of DNA repair, whereas TAM after combined with Z-LIG significantly induced DNA damage as higher levels of γ-H2AX was observed up to 24 h. In our study, we also found that the expression of Nur77 was dramatically decreased in MCF-7^TR5^ cells compared with that in MCF-7 cells (Figure 7B). Recent study suggested that Nur77 is can interact with Ku80 to repress its DNA-end binding, resulting in suppressing DSB repair [32]. To identify the potential mechanism responsible for the loss of Nur77 in MCF-7^TR5^ cells, we treated MCF-7^TR5^ cells with Z-LIG and CQ, as well as MG132, a ubiquitin-proteasome inhibitor, respectively. Our Western blotting result revealed that Z-LIG could markedly restore Nur77 expression and CQ exhibited a similar, but weaker ability, whereas MG132, couldn't recover Nur77 expression (Figure 7C). To further confirm the role of autophagy inhibition in the upregulation of Nur77 expression, we evaluated the combined action of Z-LIG with CQ or siATG6 on the Nur77 expression in MCF-7^TR5^ cells. As shown in Figure 7D, not only Z-LIG and CQ, but also siATG6 recovered Nur77 expression compared with CTRL. Moreover, the combination of two among Z-LIG, CQ and siATG6 led to a higher expression of Nur77 compared with each counterpart alone (Figure 7D). These results suggest that Nur77 protein was degraded by autophagy, but not ubiquitin-proteasome pathway. To investigate whether Nur77 plays an important role in Z-LIG- mediated DNA damage and sensitization of MCF-7^TR5^ cells. We then knocked down Nur77 protein by shRNA. Western blotting results showed that the combination of Z-LIG and TAM couldn't retain the ability to induce γ-H2AX expression in MCF-7^TR5^ cells transfected with shNur77 compared with that in MCF-7^TR5^ cells transfected with vector (Figure 7E). Then, we checked the cell viability of MCF-7^TR5^ cells treated by combinatorial Z-LIG and TAM in presence or absence of shNur77 by SRB. Similarly, the ability of Z-LIG to sensitize TAM-induced cell death was remarkably attenuated when Nur77 was knocked down (Figure 7F). There findings indicate that Nur77 played a key role in Z-LIG-mediated DNA damage and restoration of MCF-7^TR5^ cells sensitivity to TAM.

### Z-LIG restores the interaction of Nur77 with Ku80 to repress the DNA binding of Ku80

**Figure 8:**
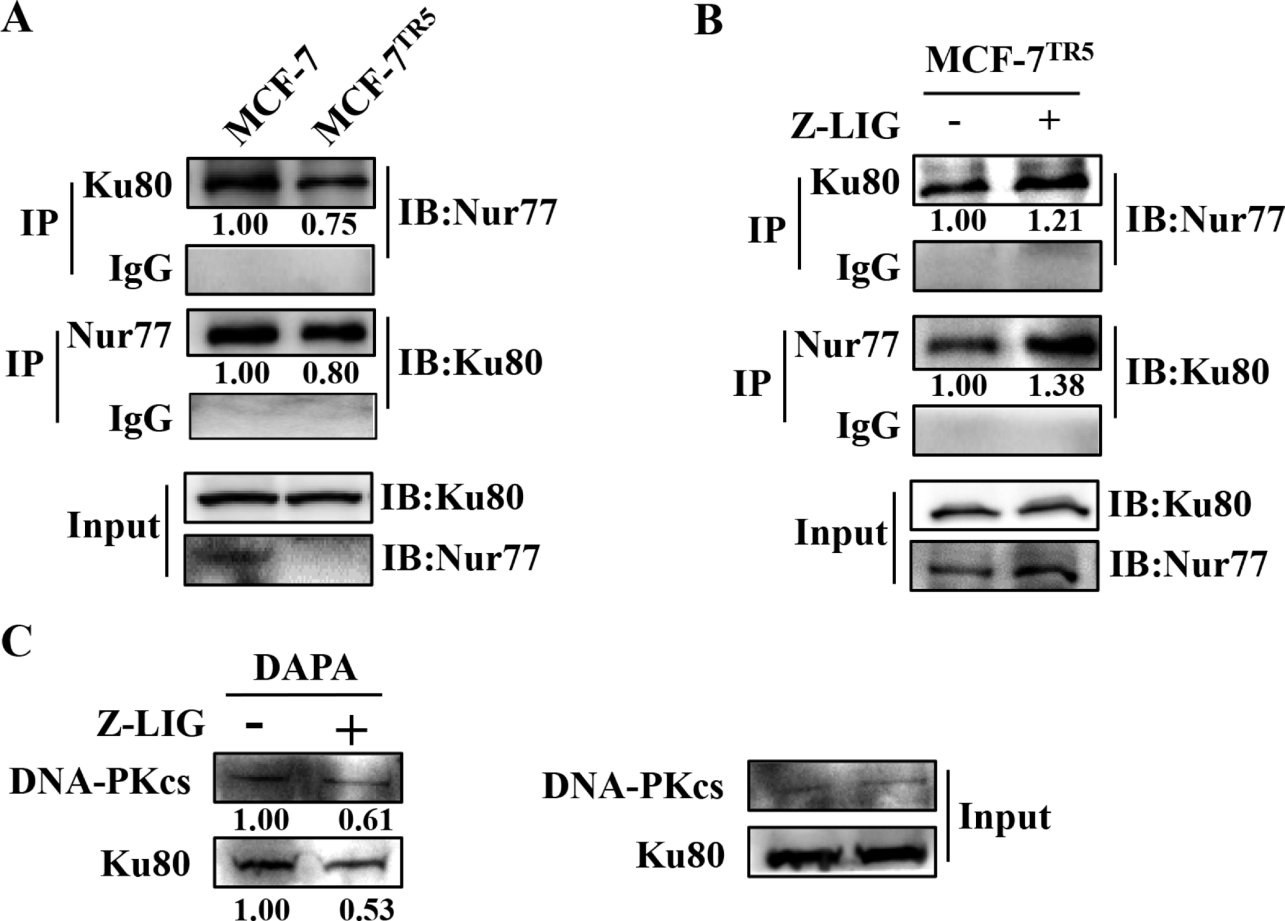
Z-LIG restores the interaction of Nur77 with Ku80 to repress the DNA binding of Ku80 (**A**) Immunoprecipitation assay of the interaction of Nur77 with Ku80 in MCF-7 and MCF-7^TR5^ cells. Cell lysate was prepared and subjected to immunoprecipitation using anti-Ku80 and anti-Nur77, respectively. Then, the associated Nur77 and Ku80 were determined using immunoblotting. (**B**) Effect of Z-LIG on the interaction of Nur77 with Ku80 in MCF-7^TR5^ cells. MCF-7^TR5^ cells were treated with or without Z-LIG (50 μM) for 24 h and then determined as (A). (**C**) Z-LIG inhibits Ku80 and DNA-PKcs binding to DNA. MCF-7^TR5^ cells were treated with or without Z-LIG (50 μM) for 24 h. Nuclear extracts were incubated either with 18-bp biotinylated double-stranded oligonucleotides for Ku80 and DNA-PKcs or streptavidin-agarose beads, but no probe, as a control and then subjected to DNA-affinity precipitation assays. Specifically bound proteins were analyzed via Western blotting using antibodies against DNA-PKcs and Ku80. The experiments were a representative of three independent experiments.

To further elucidate the exact role of Nur77 in the DNA damage repair of MCF-7^TR5^ cells and the Z-LIG-mediated increase of DNA damage, we performed coimmunoprecipitation assay to investigate the interaction of Nur77 and Ku80. As shown in Figure 8A, the interaction of Nur77 and Ku80 markedly reduced in MCF-7^TR5^ cells compared with that in MCF-7 cells. Notably, Z-LIG treatment significantly promoted the interaction between Nur77 and Ku80 in MCF-7^TR5^ cells. These results indicate that the ability of NHEJ repair in MCF-7^TR5^ cells was enhanced due to the decrease of Nur77 and Ku80 interaction, whereas this ability was impaired again after MCF-7^TR5^ cells was treated by Z-LIG, which enhanced the Nur77 and Ku80 interaction. It has been reported that the major function of Ku protein is the recruitment of DNA-PKcs to broken DNA ends, thereby releasing the catalytic potential of DNA-PK [31, 33]. We predicted that Nur77, which was recovered by Z-LIG, may affect the binding of Ku80 and DNA-PKcs to DNA ends through interacting with Ku80. To confirm our hypothesis, DNA affinity precipitation assay (DAPA) assays were performed using an end-labeled 18-bp DNA fragment as a probe. The probe efficiently pulled down endogenous Ku80 and DNA-PKcs in MCF-7^TR5^ cells (Figure 8C), suggesting that both Ku80 and DNA-PKcs form a complex with the DNA fragment in MCF-7^TR5^ cells. However, the formation of the Ku-DNA and DNA-PKcs-DNA complexes was obviously impaired by Z-LIG (Figure 8C).

## DISCUSSION

Autophagy has been observed to function as a protective and pro-survival mechanism against a number of chemotherapeutics by human cancer cell lines [15]. It has also been shown that protective autophagy is closely related to the acquired drug resistance of tamoxifen in breast cancer cells [34]. However, it remains largely unknown how autophagic flux influences the cell death triggered by tamoxifen and whether autophagy inhibitor can interfere with this process. In the current study, we aimed to clarify the potential mechanisms related to the protective autophagy in TAM-resistant breast cancer cells and characterize the inhibitory effect of Z-LIG on autophagy, as well as make clear that how Z-LIG-mediated autophagy inhibition affects the sensitivity of TAM-resistant breast cancer cells.

We first generated the stable TAM-resistant breast cancer cells MCF-7^TR5^ and T47D^TR5^, both of which are ERα^+^ breast cancer cells, with a well-established stepwise drug selection method [17]. Morphologically, TAM-resistant breast cancer cells become more bigger and round compared with TAM-sensitive breast cancer cells (data not shown). Further confirmation showed that both MCF-7^TR5^ and T47D^TR5^ cells exhibited remarkable resistant to TAM compared with their sensitive counterparts. Meanwhile, a higher level of autophagy was shown in MCF-7^TR5^ cells by the observation of GFP-LC3 puncta via fluorescent microscopy and the detection of conversion of LC3-I to LC3-II via Western blotting. Moreover, a lower p62 level was observed in MCF-7^TR5^ cells and the inhibitory effect of TAM was markedly enhanced by co-treatment of MCF-7^TR5^ cells with CQ or siATG6, which indicates that autophagy played a protective role in MCF-7^TR5^ cells. These results are consistent with the findings of previous studies demonstrating autophagy serve as a pro-survival mechanism in the tamoxifen-resistant breast cancer cells [17–20]. However, it is still largely unknown which step in autophagic flux is altered in MCF-7^TR5^ cells, resulting in the activation of pro-survival autophagy. The antiapoptotic protein Bcl-2 is well-known to interact with BH3-only domain of Beclin1, which acts as a switch to repress or activate autophagy [13]. Under normal conditions, this interaction blocks Beclin1-mediated autophagy via interfering with the formation of Beclin1-PI3KC3 complex, which is essential for the formation of autophagic vesicles. Under stress conditions, autophagy is adaptively activated upon the disruption of the binding of Beclin 1 and Bcl-2 through either stress kinases or protein-protein displacement [8]. Our co-immunoprecipitation assays revealed that the increase of autophagosome formation in MCF-7^TR5^ cells may be due to the dissociation of Bcl-2 from Beclin 1, which further results in the enhancement of interaction among the ATG14-Beclin1-PI3KC3 complex, suggesting that the alteration of Bcl-2 and Beclin 1 interaction may, at least in part, responsible for the activation of pro-survival autophagy in TAM-resistant breast cancer cells. Additionally, it is worth noting that the level of PI3KC3 and ATG14 in input increased in MCF-7^TR5^ cells, which may also contribute to the increase of interaction among ATG14, Beclin1 and PI3KC3.

Although Z-LIG has been shown to inhibit tumor necrosis factor-alpha-induced autophagy in C2C12 cells [29], nothing is yet known of the influence of Z-LIG on the autophagic flux. In this study, we first presented confirmatory evidence that Z-LIG increased the LC3-II level and RFP-LC3 puncta. Then, we further verified whether such effect was due to the increased generation of autophagosomes or a blockage in autophagosomal maturation and degradation. We found that Z-LIG enhanced the p62 protein levels and showed no additional effect on the CQ-mediated increase of LC3-II and p62 level, suggesting that Z-LIG inhibited the autophagic flux in MCF-7^TR5^ cells. During the process of autophagic flux, one critical step is the fusion of autophagosome with lysosome to form autolysosome, which then degrades the inner vesicle, together with its cargo [8, 10]. However, blockage of autophagosome-lysosome fusion also renders has been the most common mechanism by which autophagy can be inhibited during the late stage [10, 35]. In our study, Z-LIG, like CQ, was shown to block the degradation of GFP-LC3 after MCF-7^TR5^ cells were transfected with mRFP-GFP-LC3 constructs. This assay is based on the fact that GFP is unstable under the acidic condition of lysosome, while mRFP exhibits greater stable. Thus, if the autolysosome maturation progresses normally, more red-only puncta will be observed. On the contrary, if non-fused autophagosome or lysosome function is impaired, both red and green puncta will simultaneously exist, which appears to be yellow [36]. Moreover, no co-localization of GFP-LC3 and LAMP-1 was observed in MCF-7^TR5^ after Z-LIG treatment. These results presented the evidence that the process of fusion between autophagosomes and lysosomes is preferably impaired by Z-LIG. Currently, it is generally accepted that the increased acidification and cathepsin enzyme activity are the unique features of autophagosome-lysosome fusion [35]. Furthermore, our results clearly showed that Z-LIG decreased the lysosomal pH and down-regulated the cathepsin CTSD, which may be responsible for the inhibitory effect of Z-LIG on the autophagosome-lysosome fusion.

*Radix Angelica sinensis* is traditionally used for gynecological disorders in Chinese medicine. Notably, recent investigation with statistical data from Taiwan revealed that *Radix Angelica sinensis* is most frequently prescribed in herbal formula for breast cancer [22] and is taken by almost half of tamoxifen-treated breast cancer survivors [23]. These findings prompt us to consider whether the sensitivity of TAM-resistant breast cancer cells could be enhanced by the Z-LIG, a major bioactive phthalide compound in *Radix Angelica sinensis*. Our results of cell viability determination and colony formation assay indicated that Z-LIG effectively enhanced the sensitivity of TAM-resistant breast cancer cells. Moreover, our further studies showed that combinatorial Z-LIG and TAM remarkably induced a caspase-independent apoptosis of MCF-7^TR5^ cells. Consistently, previous study also demonstrated that ginsenoside Ro promotes caspase-independent cell death in 5-Fluorouracil-resistant esophageal cancer cells [37].

Recent studies have demonstrated pro-survival autophagy is closely correlated with DNA damage, which contributes to the development of drug resistance [37–39]. Thus, we determined DNA damage and the primary repair mechanisms in our cell models. Surprisingly, our results from Western blotting and immunofluorescence staining demonstrated that a higher level of γ-H2AX was observed in MCF-7^TR5^ cells compared with that in MCF-7 cells. As γ-H2AX is a well-established DSB marker, such results implicated that there may be a higher basal level of DNA damage in MCF-7^TR5^ cells. Similar results were also observed in human ovarian cancer cell lines resistant to nitrogen mustard and cisplatin [40]. In general, the DSB repair mechanisms mainly include the HR repair, an error free DNA repair process, and the NHEJ repair, an error prone DNA repair process [31]. Then, we examined the level of BRCA1 and its interacting protein RAD51, which are essential for HR repair mechanism, thus protecting the genome integrity [31]. We found that the protein level of both BRCA1 and RAD51 remarkably decreased in MCF-7^TR5^ cells, indicating a severe deficiency of HR repair mechanism in MCF-7^TR5^ cells. Furthermore, we also detected the protein level of Ku70/80, which play a key role in NHEJ repair mechanism [33]. We found that Ku80 level markedly increased in MCF-7^TR5^ cells, suggesting that NHEJ repair mechanism may be more prevalent in MCF-7^TR5^ cells. Previous studies demonstrated that deficiency of BRCA1 in cells led to a defect in the repair of DSB by the error-free mechanism of HR, which may result in the repair of such damages by error-prone mutagenic mechanisms, such as NHEJ, culminating in genomic instability [41–43]. In addition, several studies also demonstrate that BRCA1 deficiency may correlated with autophagy induction. For example, the mutation of BRCA1 can lead to mTOR inhibition [44]. Moreover, the pro-autophagic protein Beclin 1 was shown to be a target of BRCA1 [45].

Emerging evidence demonstrated that autophagy plays a protective role during DNA damage [37, 46, 47]. Thus, we determined the influence of Z-LIG-mediated autophagic flux inhibition on DNA damage. Our results indicated that Z-LIG not only counteracted the DNA repair process observed in TAM alone-treated MCF-7^TR5^ and T47D^TR5^ cells, but also enhanced the DNA damage. Recent study showed that orphan receptor protein Nur77 can interfere with the NHEJ repair pathway [32]. Interestingly, the marked loss of Nur77 protein level in MCF-7^TR5^ cells was observed in our study. Intracellular proteins and organelles can be degraded by either ubiquitin-proteasome pathway or autophagy [48]. Our results showed that the Nur77 expression was recovered by Z-LIG and CQ, as well as siATG6, but not MG132, suggesting Nur77 protein is degraded by autophagy in MCF-7^TR5^ cells and can be restored by Z-LIG-mediated autophagy inhibition. Then, we asked whether Nur77 played an important role in Z-LIG-mediated DNA damage and sensitization of TAM-resistant breast cancer cells. Expectedly, both DNA damage and inhibition on cell viability caused by combinatorial Z-LIG and TAM in MCF-7^TR5^ cells were reversed in the presence of shNur77. The NHEJ repair mechanism is initiated by the recruitment of the Ku70/80 heterodimer to DSB ends. Subsequently, NHEJ repair proteins (i.g., DNA-PKcs) are attracted and bind at both the 5′ and 3′ ends of the broken DNA to form a synaptic complex, containing the DNA ends and the repair proteins [31, 33]. Although both Ku70 and Ku80 are DNA-binding Ku proteins, our study showed that only Ku80 increased, while Ku70 decreased in MCF-7^TR5^ cells. Moreover, Nur77 is shown to only physically interact with Ku80 and thereby repress its DNA-end binding, eventually resulting in the suppression of DSB repair [32]. Thus, we first determined the interaction of Nur77 with Ku80 in MCF-7^TR5^ cells and the potential influence of Z-LIG. Our data from coimmunoprecipitation assay indicated that there may be a stronger NHEJ pathway-mediated DSB repair ability in MCF-7^TR5^ cells as the interaction of Nur77 and Ku80 remarkably decreased in MCF-7^TR5^ cells. Importantly, Z-LIG may impair this NHEJ repair mechanism as the interaction of Nur77 and Ku80 was enhanced after Z-LIG treatment. Furthermore, our DAPA assay showed that Z-LIG interfered with the DNA binding ability of both Ku80 and DNA-PKcs in MCF-7^TR5^ cells. Thus, Z-LIG-mediated impairment of DSB repair in MCF-7^TR5^ cells may be caused by the competitive binding of Nur77 to Ku80, resulting in the inhibition of Ku80 and DNA-PKcs binding at DNA-ends.

**Figure 9:**
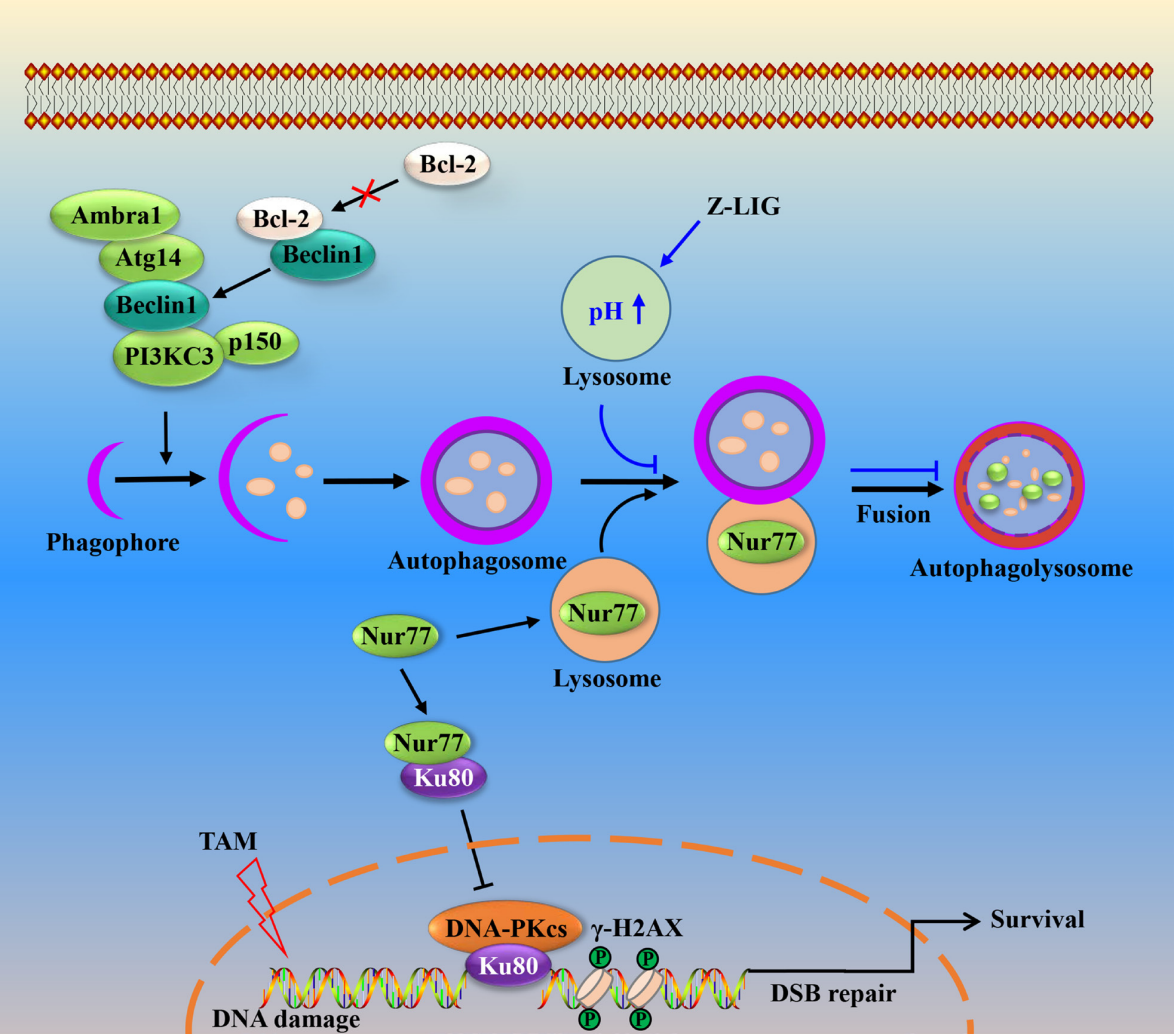
Schematic illustration of Z-LIG inhibiting autophagic flux and sensitizing TAM-resistant breast cancer cells to cell death In MCF-7^TR5^ cells, protective autophagy is activated partly through the dissociation of Bcl-2 from Beclin 1 and the increased interaction of ATG14-Beclin1-class III PI3K. Meanwhile, Nur77 is selectively degraded by autophagy, leading to the disruption of Nur77 and Ku80 interaction. Subsequently, Ku80 binds to the DNA ends and recruits DNA-PKcs to complete the DSB repair via NHEJ. When the fusion process of autophagosome and lysosome is blocked by Z-LIG via altering lysosomal pH and downregulating lysosomal cathepsin, Nur77 is restored and then directly bind to Ku80, thereby abolishing the association of DNA-PKcs with DNA ends, which suppresses DSB repair. Consequently, the sensitivity of MCF-7^TR5^ cells to TAM is enhanced by Z-LIG in an apoptosis-independent and autophagy-associated way.

Taken together, the findings we present in this study mechanistically connect autophagy and DNA repair to provide the explanation for the formation of TAM resistance in breast cancer cells and the sensitization effect of Z-LIG (Figure 9). We show that the dissociation of Bcl-2 from Beclin 1 and subsequent enhancement of interaction among the ATG14-Beclin1-PI3KC3 complex lead to the induction of protective autophagy in TAM-resistant breast cancer cells. Moreover, the defect in the repair of DSB by HR may result in the high basal level of DNA damage in MCF-7^TR5^ cells. Alternatively, NHEJ pathway is compensatively induced to repair DNA damage in MCF-7^TR5^ cells through the selective degradation of Nur77 by autophagy, which leads to the release of Ku80 from the binding with Nur77, resulting in the increase of the DNA binding ability of Ku80 and DNA-PKcs. Another important finding in our study is that Z-LIG inhibits autophagic flux through the blockage of the autophagosome-lysosome fusion. Importantly, Z-LIG-mediated autophagy inhibition restores Nur77 expression in MCF-7^TR5^ cells, which promotes the interaction of Nur77 with Ku80, thereby abolishing the association of DNA-PKcs with DNA ends and subsequently suppressing DSB repair. Ultimately, Z-LIG sensitizes TAM-resistant breast cancer cells in a caspase-independent apoptosis. Thus, we provided the potential mechanisms for the formation of TAM resistance in breast cancer cells. Moreover, we identified Z-LIG as novel autophagy inhibitor to enhance TAM-based breast cancer therapy.

## MATERIALS AND METHODS

### Materials

Z-LIG with purity more than 98% was obtained from Chengdu Must Bio-Technology Co, Ltd (Chengdu, China) and stored in −80 °C before use. The antibodies against BRCA1, Nur77, Bcl-2 and p62 were purchased from Santa Cruz Biotechnology (CA, USA). PI3KC3 and PARP antibodies were obtained from Cell Signaling Technology (Boston, MA, USA). The antibodies against CTSB and CTSD were obtained from Wanlei Biotechnology (Shenyang, China). The antibodies against ATG14, Beclin1, Ku70/80 and DNA-PKcs were purchased from Proteintech Group Inc (Wuhan, China). The antibodies against γ-H2AX and Rad51 were obtained from Bioss Biotechnology (Beijing, China). The antibodies against LC3, β-actin and rabbit IgG were obtained from Sigma-Aldrich (St. Louis, MO, USA). Other chemicals were obtained from Sigma-Aldrich, unless indicated otherwise.

### Cell culture

Human breast cancer cells MCF-7 and T47D cells were obtained from the American Type Cell Culture Collection (Manassas, VA). All the cells were maintained in Dulbecco's modified Eagle's medium (DMEM) supplemented with 10% fetal bovine serum (FBS) (Invitrogen, USA) and 1% penicillin/streptomycin (Invitrogen, USA) at 37°C in a humidified 5% CO2 atmosphere and seeded in triplicate for all experiments. TAM-resistant MCF-7 and T47D cells were selected according to previous study [17]. Briefly, MCF-7 and T47D cells were exposed to small, incremental increases of TAM (1, 2, 3, 4, and 5 μM) to generate TAM-resistant sublines.

### Measurement of cell viability

Cell viability was evaluated by sulforhodamine B (SRB) assay (Sigma-Aldrich, St. Louis, MO, USA), which was based on the measurement of cellular protein content and stained with 0.4% SRB for 30 min. The protein-bound dye is dissolved in 10 mM Tris base solution for OD determination at a wavelength of 490 nm using a multi-well spectrophotometer microplate reader (Biotek, Winooski, VT, USA). Cell viability was expressed as a percentage of that of the control (untreated) cells.

### Plasmids transfection

The GFP-LC3, RFP-LC3, RFP-LAMP1 and mRFP-LC3 tandem fluorescence-tagged LC3 construct (tfLC3) were provided by Addgene. MCF-7 and MCF-7^TR5^ cells were transfected with GFP-LC3B or RFP-LC3 plasmid using lipofectamine 2000 (Invitrogen, 11668–019). After a 6 hour incubation, the transfection medium was removed, and the cells were incubated in fresh medium for 24 h. Then, cells were treated with TAM (5 μM) or Z-LIG (50 μM) for 24 h prior to fixation. Image acquisition was done using an Olympus FV1000 (Olympus) confocal microscope.

### Western blotting

The cellular proteins were extracted from MCF-7, MCF-7^TR5^, T47D and T47D^TR5^ cells in ice-cold RIPA buffer (Cell Signaling Technologies, USA) supplemented with 1% (v/v) protein inhibitor cocktail and 1 mM phenylmethylsulfonyl fluoride (PMSF). Cell extracts were resolved by SDS-PAGE and transferred to a polyvinylidene difluoride (PVDF) membrane. Following 1 h incubation in a fresh TBS buffer containing 0.1% Tween-20 and 5% BSA, the membranes were probed with the corresponding primary antibodies. Following incubation with horseradish peroxidase coupled secondary antibodies. Then, protein bands were visualized using an enhanced chemiluminescence (ECL) detection reagent (GE Healthcare, Sweden). The concentration of the loaded cellular proteins was normalized against the internal control β-actin, and then the value was expressed as each normalized data relative to control.

### RNA interference

Small interfering RNAs targeting Atg6 (siAtg6) and a control siRNA (si-CTRL) were obtained from sigma (St. Louis, MO, USA). Short hairpin RNAs targeting Nur77 (shNur77) and a control shRNA (shCTRL) were obtained from YRgene. Cells were then transfected with 50 nM siRNA or 3 μg shRNA using transfection reagent Lipofectamine 2000 (Invitrogen, USA) according to the manufacturer's instructions. After a 6 h antibiotic-free medium incubation, the transfection medium was removed, and the cells were incubated in fresh medium for 24 h, followed by further drug treatments.

### Immunoprecipitation

MCF-7 and MCF-7^TR5^ cells were lysed with ice-cold RIPA buffer (Cell Signaling Technologies, USA) supplemented with 1% (v/v) protein inhibitor cocktail and 1 mM PMSF. Primary antibody was covalently immobilized on protein A/G agarose provided by Beyotime (Jiangsu, China) according to the manufacturer's instructions. Samples were incubated with immobilized antibody beads for at least 1 h to 3 h in 4°C. After that, the samples were washed with TBS five times. They were then incubated in the boiling water bath for 3 to 5 minutes and the complexes recovered on beads were analyzed by Western blotting.

### RNA isolation and quantitative RT-PCR

Total RNA isolation was performed using Trizol reagent (Invitrogen, USA) following the manufacturer's protocol. Reverse transcription PCR was done using PrimeScript RT reagent kit (TaKaRa, DRR037A). Primers for qPCR reactions were as follows: p62 (human): (F) 5′-GAACTCCAGTCCCTACAGATGCC-3′, (R) 5′-CG GGAGATGTGGGTACAAGG-3′; GAPDH (human): (F) 5′-TGTTGCCATCAATGACCCCTT-3′, (R) 5′-CTCCACG ACGTACTCAGCG-3′.

### Acridine orange staining

After treatment with Z-LIG or CQ, cells were washed twice with PBS, followed by incubation with 0.005 μg/ml acridine orange (Invitrogen) at 37°C for 15 min. After washing with PBS 3 times, the fluorescence signal was detected and recorded under a Zeiss fluorescence microscope (Carl Zeiss, Germany).

### Hoechst 33342 staining

After treatment at 12-well plates for 48 h, cells were washed with PBS for 3 times. Then, Hoechst 33342 (Wanlei Biotechnology, Shengyang, China) dissolving in PBS was added into each well. The plates were kept at room temperature for 10 min and avoided from light. Finally, the plates were washed with PBS again and images were captured on a Zeiss fluorescence microscope (Carl Zeiss, Germany).

### Colony formation assay

MCF-7^TR5^ cells were seeded in a 6-well plate and treated with Z-LIG (50 μM), TAM (2.5 μM) or combination with Z-LIG and TAM before reseeded in six-well plates (3,000 cells/ well), respectively. Colonies were allowed to form for two weeks and medium with drugs or vehicle was replaced per 3 days. At the end of treatment, cells were fixed in 100% methanol and stained with 0.005% crystal violet. Finally, images were captured by a CCD camera and the colonies were counted.

### γ-H2AX immunofluorescence staining

MCF-7 and MCF-7^TR5^ cells were seeded to 12-well plates and TAM (5 μM) treated for 24 h. Cells were washed 2 times with PBS, then fixed with 4% paraformaldehyde in PBS for 15 min at room temperature, and permeabilization with 0.25% Triton X-100 in PBS for 10 min at 4°C. Cells were washed with PBS and blocked with 5mg/ml BSA in PBS for 1 h, then incubated with rat anti-rabbit γ-H2AX primary antibody in a 1:100 dilution overnight at 4°C, followed by FITC secondary antibody (Beyotime). Photos was observed by Zeiss fluorescence microscope (Carl Zeiss, Germany).

### DNA affinity precipitation assay

DAPA were performed as described previously [32]. Nuclear extracts (200 μg) were mixed with 2 μg specific, biotinylated double-stranded DNA probes (forward, 5-AGGCTGTGTCCTCAGAGG-3; reverse, 5-CCTCTGAGGACACAGCCT-3) and 10 μg poly-deoxyinosine-deoxycytosine in 500 μl DAPA buffer for 4 h at 4°C. Next, 20 μl streptavidin-agarose beads (Invitrogen, Carlsbad, CA) was added, and the samples were agitated for 2 h at 4°C. The agarose bead-protein complexes were boiled in water bath for 3 to 5 minutes. The protein-DNA probe complexes recovered on beads were analyzed via Western blotting.

### Statistical analysis

All data were presented as mean ± SD for three independent experiments. A ANOVA test was used to calculate the significant difference in the study. A *p*-value of less than 0.05 was considered to be statistically significant.

## SUPPLEMENTARY MATERIALS FIGURES



## Abbreviations

ERα: Estrogen receptor alpha
TAM: Tamoxifen
Z-LIG: Z-ligustilide
ERα^+^: estrogen receptor alpha positive
TR5: TAM resistant to 5 μM
PI3KC3: Class III PI3K
CQ: chloroquine
AO: acridine orange
SRB: Sulforhodamine B
DSB: double-strand break
NHEJ: non-homologous end-joining
HR: homologous recombination
