# Supplementary material for: Sensitization of tamoxifen-resistant breast cancer cells by Z-ligustilide through inhibiting autophagy and accumulating DNA damages
Source: Oncotarget. 2017 Apr 4;8(17):29300–17. doi: 10.18632/oncotarget.16832 (PMC5438731; doi:10.18632/oncotarget.16832)
Supplement: Supplementary file 1 [file oncotarget-08-29300-s001.pdf]

Sensitization of tamoxifen-resistant breast cancer cells by Z-ligustilide through inhibiting autophagy and accumulating DNA damages

Supplementary Materials

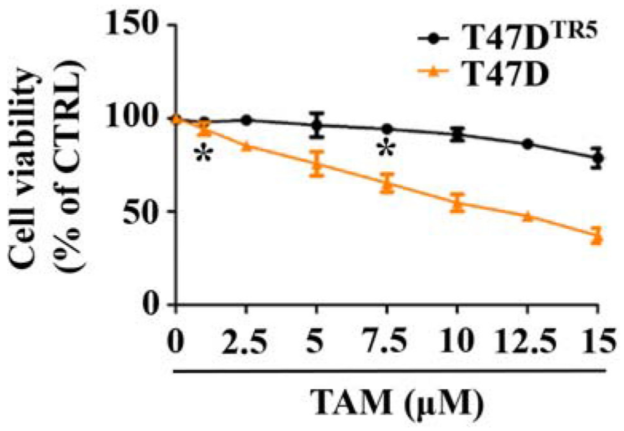

Supplementary Figure 1: Effect of TAM on the cell viability of T47D and T47D[TR5] cells. Cells were treated by TAM as indicated for 72 h and cell viability were determined by SRB assay. Values represent mean±SD. *$p<0.05$.

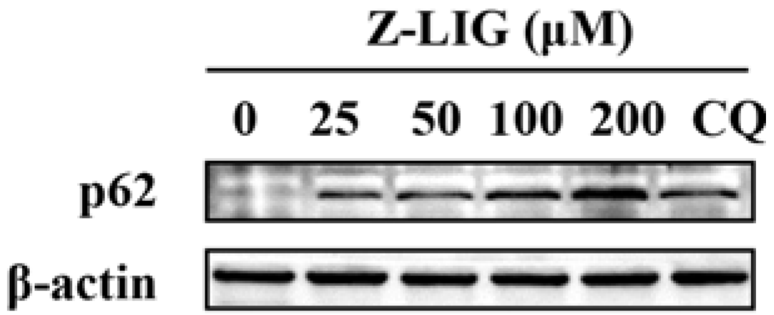

Supplementary Figure 2: Effect of Z-LIG on p62 expression in T47D[TR5] cells. Cells were treated with Z-LIG with indicated concentrations for 24 h. Then, the expression of p62 was analyzed by Western blotting. The blots were a representative of three independent experiments.

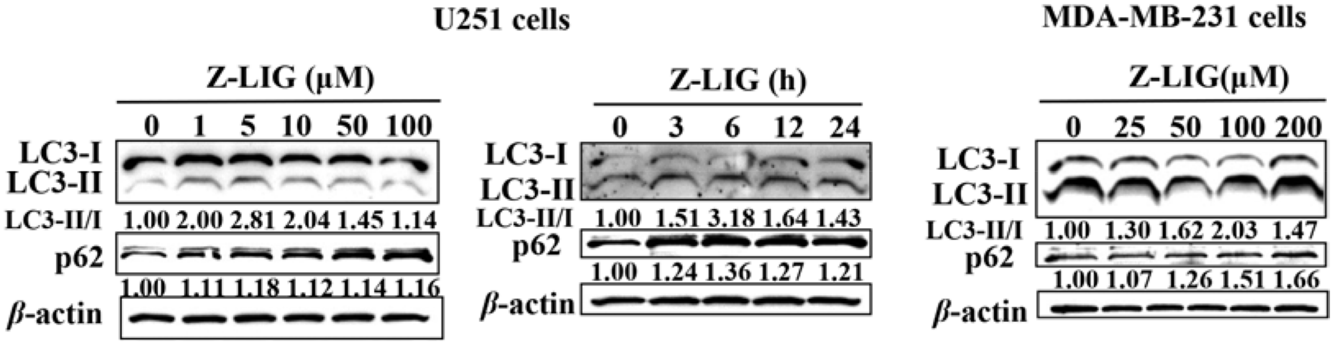

Supplementary Figure 3: Effect of Z-LIG on conversion of LC3II and p62 expression in U251 cells and MDA-MB-231 cells. U251 cells were treated with Z-LIG with indicated concentrations for 24 h or treated with Z-LIG (50 μM) for indicated time points. MDA-MB-231 cells were treated with Z-LIG with indicated concentrations for 24 h. Then, the expression of LC3II/I and p62 was analyzed by Western blotting.

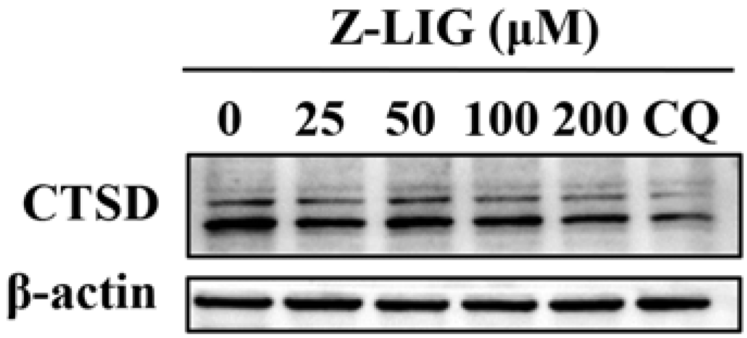

Supplementary Figure 4: Effect of Z-LIG on the expression of CTSD protein. T47D cells were treated with Z-LIG as indicated or 20 µM CQ for 24 h, and the expression of CTSD protein was determined by Western blotting.

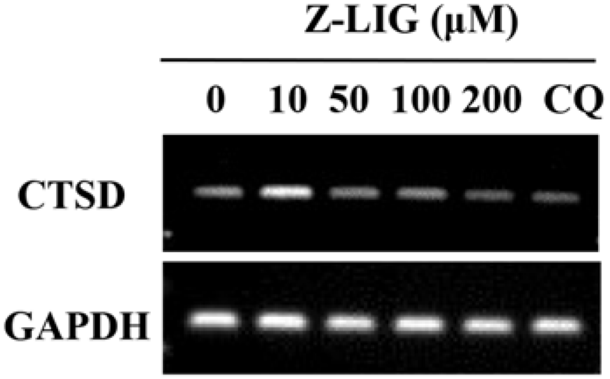

Supplementary Figure 5: Effect of Z-LIG on the expression of CTSD mRNA. MCF-7 cells were treated with Z-LIG as indicated or 20 µM CQ for 24 h, and the expression of CTSD mRNA were determined by RT-PCR.

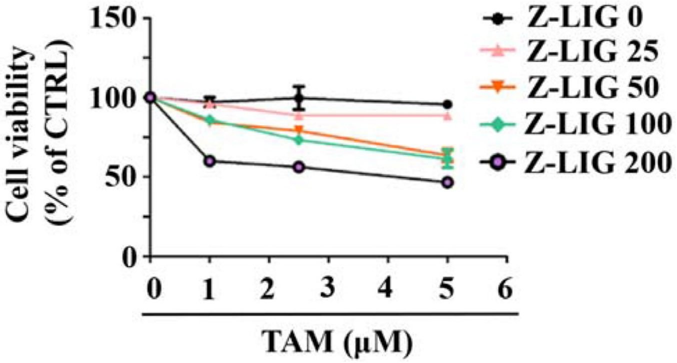

Supplementary Figure 6: Effect of combinatorial Z-LIG and TAM on cell viability. T47D^{TR5} cells were pretreated with Z-LIG as indicated for 12 h and then treated with TAM as indicated for 72 h. The cell viability was determined by SRB assay. Values represent mean ± SD.

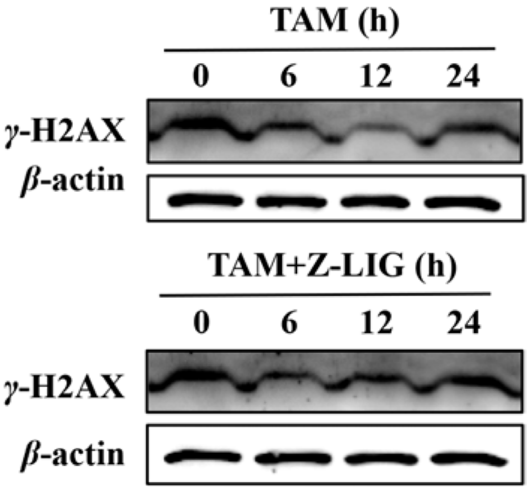

Supplementary Figure 7: Effect of TAM or combinatorial TAM and Z-LIG on expression of γ-H2AX. T47D^{TR5} cells were pretreated with or without Z-LIG (50 μM) for 12 h and then exposed to TAM (5 μM) for 0, 6, 12 and 24 h. The expression of γ-H2AX was determined by Western blotting. The blots were a representative of three independent experiments.
